# A Functional Metagenomic Analysis of Tetracycline Resistance in Cheese Bacteria

**DOI:** 10.3389/fmicb.2017.00907

**Published:** 2017-05-24

**Authors:** Ana B. Flórez, Lucía Vázquez, Baltasar Mayo

**Affiliations:** Departamento de Microbiología y Bioquímica, Instituto de Productos Lácteos de Asturias – Consejo Superior de Investigaciones Científicas, IPLA – CSICAsturias, Spain

**Keywords:** antibiotic resistance, resistance genes, tetracycline resistance, cheese, traditional cheeses, culture-independent methods

## Abstract

Metagenomic techniques have been successfully used to monitor antibiotic resistance genes in environmental, animal and human ecosystems. However, despite the claim that the food chain plays a key role in the spread of antibiotic resistance, metagenomic analysis has scarcely been used to investigate food systems. The present work reports a functional metagenomic analysis of the prevalence and evolution of tetracycline resistance determinants in a raw-milk, blue-veined cheese during manufacturing and ripening. For this, the same cheese batch was sampled and analyzed on days 3 and 60 of manufacture. Samples were diluted and grown in the presence of tetracycline on plate count milk agar (PCMA) (non-selective) and de Man Rogosa and Sharpe (MRS) agar (selective for lactic acid bacteria, LAB). DNA from the cultured bacteria was then isolated and used to construct four fosmid libraries, named after the medium and sampling time: PCMA-3D, PCMA-60D, MRS-3D, and MRS-60D. Clones in the libraries were subjected to restriction enzyme analysis, PCR amplification, and sequencing. Among the 300 fosmid clones analyzed, 268 different EcoRI restriction profiles were encountered. Sequence homology of their extremes clustered the clones into 47 groups. Representative clones of all groups were then screened for the presence of tetracycline resistance genes by PCR, targeting well-recognized genes coding for ribosomal protection proteins and efflux pumps. A single tetracycline resistance gene was detected in each of the clones, with four such resistance genes identified in total: *tet*(A), *tet*(L), *tet*(M), and *tet*(S). *tet*(A) was the only gene identified in the PCMA-3D library, and *tet*(L) the only one identified in the PCMA-60D and MRS-60D libraries. *tet*(M) and *tet*(S) were both detected in the MRS-3D library and in similar numbers. Six representative clones of the libraries were sequenced and analyzed. Long segments of all clones but one showed extensive homology to plasmids from Gram-positive and Gram-negative bacteria. *tet*(A) was found within a sequence showing strong similarity to plasmids pMAK2 and pO26-Vir from *Salmonella enterica* and *Escherichia coli*, respectively. All other genes were embedded in, or near to, sequences homologous to those of LAB species. These findings strongly suggest an evolution of tetracycline resistance gene types during cheese ripening, which might reflect the succession of the microbial populations. The location of the tetracycline resistance genes in plasmids, surrounded or directly flanked by open reading frames encoding transposases, invertases or mobilization proteins, suggests they might have a strong capacity for transference. Raw-milk cheeses should therefore be considered reservoirs of tetracycline resistance genes that might be horizontally transferred.

## Introduction

Some 90 years after the discovery of antibiotics and the treatment of the first patients, antibiotic resistance in bacteria -a consequence of the use, misuse and overuse of these compounds (Woolhouse et al., 2016)- has become a major problem for human health. Most studies on antibiotic resistance have naturally focused on the types of resistance shown by pathogenic and opportunistic microorganisms (Rodriguez-Rojas et al., 2013; Fair and Tor, 2014); much less attention has been paid to antibiotic resistance in commensal and beneficial bacteria (Wang et al., 2012; Broaders et al., 2013). Antibiotic resistance is, however, no longer understood as a purely clinical issue since resistance genes are known to settle into mobile genetic elements with inter-species transfer capability (Brown-Jaque et al., 2015). Indeed, antibiotic resistance determinants have been found in commensal and beneficial microorganisms that are identical to those present in pathogens, supporting the idea of the existence of a pool of shared resistance genes (Martinez, 2009; Forsberg et al., 2012). The prevalence of antibiotic-resistant bacteria in a broad range of foods (Wang et al., 2012) has led to growing concern about the impact that food (and hence food-borne bacteria) may have as a reservoir of genes involved in the spread of antibiotic resistance (Marshall and Levy, 2011). The complex microbial interactions that take place during the manufacture and ripening of fermented food products (Irlinger and Mounier, 2009), and the following contact of these microorganisms with the microbial populations of the gastrointestinal tract (Qin et al., 2010), provide ideal opportunities for horizontal gene transfer.

Recent years have seen the characterization of resistance determinants in a number of antibiotic-resistant bacteria isolated from dairy products (Ammor et al., 2007; Devirgiliis et al., 2010; Soares-Santos et al., 2015) and other food matrices (Devirgiliis et al., 2010; Li et al., 2011; Flórez et al., 2014; Flórez and Mayo, 2015), but the dairy “resistome” -i.e., all the antibiotic resistance determinants present in dairy products- is far from fully known. Metagenomic analysis and massive sequencing techniques have been used to monitor antibiotic resistance genes in environmental (Hatosy and Martiny, 2015), animal (Kazimierczak et al., 2009), and human ecosystems (Diaz-Torres et al., 2003; Seville et al., 2009; Mullany et al., 2012; Forslund et al., 2014), and would appear promising as means of characterizing the same in dairy environments. These techniques allow for the molecular characterization of the resistance genes and their associated genetic elements; this may further help to estimate the risk for their horizontal transference. However, sequencing strategies alone are neither useful for addressing the functionality of the genes discovered nor, and most importantly, for the discovery of yet unknown antibiotic resistance determinants, questions that can be addressed by a functional metagenomics approach (Kazimierczak et al., 2009). Functional metagenomic analysis has already been used to screen for antibiotic resistance genes in Mozzarella cheese (Devirgiliis et al., 2014). Additional metagenomic studies on a wider variety of cheeses and other dairy products might allow the resistome of dairy ecosystems to be determined.

The antibiotic tetracycline could be used to control a wide range of Gram-positive and Gram-negative bacterial infections (Grossman, 2016). Unfortunately, it was once also widely used as a prophylactic and growth promoter in the stock raising, fish farming and agricultural sectors (Grossman, 2016), the legacy of which is the high number of tetracycline-resistant bacteria now found in different environments, including food (Thaker et al., 2010; Grossman, 2016).

The present work reports the prevalence and evolution of tetracycline resistance determinants in a traditional, blue-veined, Spanish cheese made from raw milk, as determined by functional metagenomics.

## Materials and Methods

### Microbial Counts, Bacterial Isolates, and Growth Conditions

Ten-fold dilutions (prepared in Ringer’s solution [(VWR International]) were made of homogenized samples of Cabrales cheese (a raw-milk, blue-veined, Spanish cheese, produced with no starter culture) at day 3 (early manufacture) and day 60 (ripening) of production. These dilutions were plated, in duplicate, on plate count milk agar (PCMA -a non-selective medium) (VWR International), in the presence and absence of tetracycline (25 μg mL^-1^) for the enumeration of total aerobic mesophilic bacteria. Lactic acid bacteria (LAB) were similarly enumerated on Man, Rogosa and Sharpe (MRS) agar (VWR International), with and without the same antibiotic. All plates were incubated at 32°C for 48 h under aerobic conditions. *Escherichia coli* was grown on liquid or solid Luria–Bertani (LB) medium at 37°C with shaking.

### Extraction of High Molecular Weight Bacterial DNA

Tetracycline-resistant cells showing semi-confluent colonies on the counting plates were harvested, washed in sterile phosphate-buffered saline (PBS), and stored until use at –80°C in brain heart infusion broth (BHI) (Merck) supplemented with 25% glycerol (stock suspensions). DNA from culturable tetracycline-resistant bacteria was extracted from 180 μl of cell stock suspensions. Cell pellets were collected by centrifugation and suspended in the same volume of a lysis buffer containing 20 mg mL^-1^ lysozyme, 200 U mutanolysin, 50 μg mL^-1^ lysostaphin, 20 mM Tris-HCl (pH 8.0), 2 mM EDTA, and 1.2% Triton-X-100 (Sigma–Aldrich). The lytic suspension was then incubated at 37°C for 1 h and total DNA extracted using the DNeasy Blood and Tissue kit (Qiagen) according to the manufacturer’s protocol. The DNA yield achieved from the tetracycline-resistant bacterial suspensions was ≈0.5 μg μL^-1^. DNA with 5′ and/or 3′ protruding ends was converted to 5′-phosphorylated, blunt-ended DNA using the DNA End-Repair kit (Epicentre). The size of the end-repaired DNA was then compared by electrophoresis in low melting point agarose (Pronadisa) with that of fosmid control DNA (Epicentre). The band containing the DNA fraction between 30 and 40 kilobase pairs (kbp) was excised from the gel, the agarose degraded using the GELase enzyme (Epicentre), and the DNA purified by phenol/chloroform extraction. Finally, the DNA was precipitated by the addition of a 1/10 volume of 3 M sodium acetate (pH 7.0) and one volume of isopropanol (Merck). It was then washed in 70% ethanol, suspended in TE buffer (10 mM Tris-Cl, 1 mM EDTA, pH 8.0), and stored at –20°C until use.

### Construction of Fosmid Metagenomic Libraries

For library construction, *Escherichia coli* EPI300-T1^R^ (phage T1-resistant) (Epicentre) was grown on LB supplemented with 10 mM MgSO_4_ and 0.2% maltose, at 37°C for 24 h. Metagenomic libraries were constructed using the CopyControl^TM^ Fosmid Library Kit (Epicentre) following the supplier’s recommendations. Briefly, 0.25 μg of end-repaired DNA (∼30–40 kbp in size) was ligated with Fast-Link DNA ligase (Epicentre) to 0.5 μg of the fosmid pCC1FOS vector (Epicentre), linearized at the unique Eco72I site, and dephosphorylated. The ligation reaction was heat inactivated and used for phage packaging at 30°C for 2 h in MaxPlax Lambda Packaging extracts (Epicentre). *E. coli* cells harboring fosmid clones were selected with chloramphenicol (12.5 μg mL^-1^) (Sigma–Aldrich) and preserved in LB broth and 20% glycerol at –80°C in 96 multi-well plates (≈100 cells/well). The libraries were then replicated on LB medium supplemented with 10 μg mL^-1^ tetracycline (Sigma-Aldrich). The phage particle titre per library was determined prior to plating using the following formula:

(1) $$\begin{array}{c} T\;=\;\frac{\left(\mathrm{no}.\mathrm{colonies})\;\times \;(\mathrm{dilution}\;\mathrm{factor})\;\times \;(1,000\;\mu l/ml\right)}{\left(\mathrm{volume}\mathrm{of}\mathrm{phage}\mathrm{plated}\mathrm{in}\mu l\right)}\;\left(1\right) \end{array}$$

### DNA Extraction from Fosmid Clones, and Screening for Tetracycline Resistance Genes

A total of 300 fosmid clones phenotypically resistant to tetracycline were selected to characterize the tetracycline resistance genes they harbored. DNA from the fosmid clones was first isolated according to standard procedures (Sambrook and Russell, 2001). To increase the extraction yield, 1xCopy Number Induction Solution (Epicentre) was added to the *E. coli* cultures before extraction, according to the manufacturer’s instructions. DNA from the fosmids was then subjected to restriction digestion analysis with the endonuclease EcoRI (Takara). The DNA fragments were separated by electrophoresis in 2% agarose gels, stained with ethidium bromide (0.5 mg mL^-1^), and visualized under UV light. For sequencing, the DNA of selected fosmid clones was isolated using the commercial QIAprep Miniprep Kit (Qiagen).

The presence of tetracycline resistance genes was investigated by PCR using the universal primers DI-DII (Clermont et al., 1997) and Tet1-Tet2 (Barbosa et al., 1999) for genes encoding ribosomal protection proteins, and specific primers for the *tet*(W) (Scott et al., 2000) *tet*(M), *tet*(S), *tet*(O), *tet*(K), and *tet*(L) genes (Gevers et al., 2003b). The PCR conditions used for each primer pair were those described by the cited articles. Amplicons were sequenced in an ABI 373 DNA sequencer (Applied Biosystems) and the sequences obtained compared with those in the NCBI database using Blast software^1^.

### Sequencing of Fosmid Clones, Assembly, and Annotation

Tetracycline resistant fosmid clones with different restriction patterns were sequenced at one end using the pCC1R primer (Epicentre) and the sequences compared as above. DNA from six representative fosmids was used to construct short insert genomic libraries of 0.5 kbp, which were then subjected to 91 bp paired-end sequencing using a HiSeq 2000 System sequencer (Illumina), providing a coverage of greater than 300-fold. Quality-filtered reads were assembled in contigs using Velvet software v.1.2.10^2^. To close the sequence gaps, primers based on sequences at the extremes of the contigs were designed and used in PCR amplification. The amplicons were then sequenced. The Vector NTI program (Invitrogen) was used to assemble the contigs and PCR-derived sequences, and to predict putative open reading frames (ORFs). Predicted ORFs and deduced protein sequences were independently examined for homology against the non-redundant NCBI databases as described above.

### Antibiotic Resistance/Susceptibility Patterns of Fosmid Clones

The minimum inhibitory concentrations (MICs) of 16 antibiotics (gentamicin, kanamycin, streptomycin, neomycin, tetracycline, erythromycin, clindamycin, chloramphenicol, ampicillin, penicillin G, vancomycin, quinupristin–dalfopristin, linezolid, trimethoprim, ciprofloxacin, and rifampicin) for *E. coli* EPI300-T1^R^ harboring tetracycline resistant fosmid clones were determined using VetMIC plates (National Veterinary Institute of Sweden). Briefly, individual colonies of clones in *E. coli* were grown on LB agar plates supplemented with 10 μg mL^-1^ tetracycline and then suspended in 2 mL sterile saline solution (Oxoid) to obtain a density corresponding to that of McFarland standard 1 (≈3 × 10^8^ cfu mL^-1^). The suspension was further diluted 1:1000 in LB (final concentration 3 × 10^5^ cfu mL^-1^), and then 100 μL of this dilution was added to each well of the VetMIC plates. MICs were scored as the lowest antibiotic concentration at which no visible growth was observed after 24 h of incubation at 37°C.

### Nucleotide Sequence Accession Numbers

The DNA sequences for the six fosmid clones described above were deposited in the GenBank database under accession numbers KY686299 through KY686304.

## Results

### Total and Tetracycline-Resistant Bacterial Counts

In the absence of tetracycline, mean counts of 3.5 × 10^9^ and 1.1 × 10^7^ cfu g^-1^ cheese were scored on PCMA for total aerobic mesophilic bacteria at day 3 and day 60, respectively. The presence of tetracycline caused a reduction of around 1.0 Log_10_ unit, with final numbers of 6.1 × 10^8^ and 4.1 × 10^6^ cfu g^-1^ cheese recorded on day 3 and day 60, respectively. LAB counts on MRS agar plates at day 3 were 3.6 × 10^6^ in the absence of the antibiotic and 1.5 × 10^5^ cfu g^-1^ in its presence. At day 60, however, the numbers of LAB in the absence and presence of tetracycline were much more similar, at 3.2 × 10^6^ and 2.4 × 10^6^ cfu g^-1^, respectively.

### Metagenomic Libraries

Four independent libraries were constructed in the pCC1FOS fosmid-vector using total DNA from tetracycline-resistant bacteria growing on the PCMA and MRS agar plates at day 3 and day 60 of manufacture. These were named PCMA-3D, PCMA-60D, MRS-3D, and MRS-60D. The estimated number of clones obtained for the libraries was 3.6 × 10^5^ and 8.5 × 10^5^ cfu mL^-1^ for the PCMA-3D and PCMA-60D libraries, respectively, and 4.1 × 10^4^ and 5.2 × 10^3^ cfu mL^-1^ for the MRS-3D and MRS-60D libraries, respectively. Fosmid clones from the libraries were then replicated on LB medium supplemented with 10 μg mL^-1^ tetracycline, revealing all to confer resistance upon the *E. coli* host strain. Restriction digestion analysis of 300 clones with EcoRI showed an average insert size for the clones of around 35 kbp. Assuming a mean bacterial genome size of 4 Mb, these fosmid libraries therefore contained (on average) about 3,000 bacterial genomes each.

**FIGURE 1:**
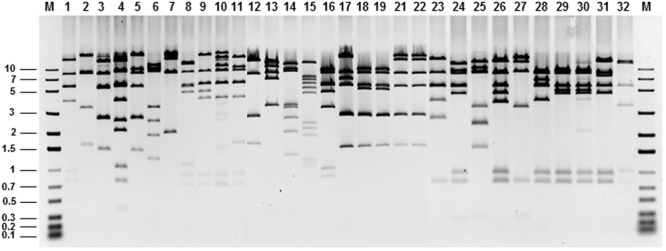
**Agarose gel electrophoresis of purified DNA from 32 tetracycline resistance fosmid clones digested with EcoRI showing diversity of the digestion patterns.** M, molecular weight marker (in kpb).

**FIGURE 2:**
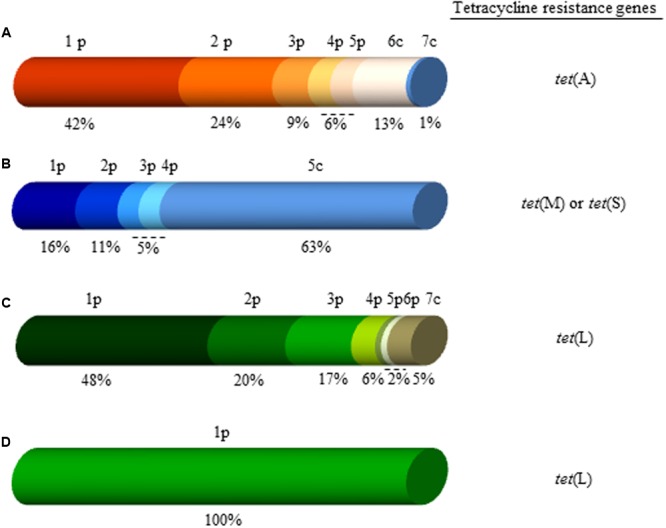
**Diagram grouping the tetracycline-resistant clones from the four fosmid libraries constructed in this work according to the homology of their end-reads to those in databases.** On top, the elements with the highest homology (see below) are indicated, and below their relative percentages. Also, indicated, the tetracycline resistance genes detected in each of the libraries (on the right). Small letter type p and c stand for plasmid or chromosomal sequences, respectively. **(A)** PCMA-3D library: 1p, *Escherichia coli* pO26-Vir; 2p, *E. coli* pHNFP460; 3p, *E. coli* pC59-153; 4p, *E. coli* pYDC637; 5p, plasmidic sequences from other enterobacteria; 6c, chromosomal sequences from enterobacterias, and 7c, chromosomal sequences from *Lactococcus lactis*. **(B)** MRS-3D library: 1p, *L. lactis* pEps352; 2p, *L. lactis* subsp. *lactis* pIBB-JZK; 3p, *Enterococcus faecium* p5753cB; 4p, *Leuconostoc carnosum* pKLC4, and 5c, chromosomal sequences from *L. lactis*. **(C)** PCMA-60D library: 1p, *Enterococcus faecalis* pJH1; 2p, *E. faecalis* pRE25; 3p, *E. faecium* pF856; 4p, *E. faecium* p1; 5p, *L. lactis* pVF18; 6p, *E. faecium* pVF4, and 7c, chromosomal sequences from *Staphylococcus* spp. **(D)** MRS-60D library: 1p, *E. faecium* pF856. The same color and tone denote identical sequences.

Among the 300 tetracycline-resistant clones assayed by EcoRI digestion, 268 restriction profiles were encountered (**Figure 1** shows partial results for 32 clones). All these clones were selected for sequencing of their extremes with pCC1FOS-specific primers. Analysis of the sequences revealed the majority to show homology to mobile genetic elements from Enterobacteriaceae species, or to plasmids from *Enterococcus faecium, Enterococcus faecalis*, or *Lactococcus lactis* (**Figure 2**). About 87% of end-read sequences from the PCMA-3D library (**Figure 2A**) were identical to others from *E. coli* plasmids, including pO26-Vir, pHNFP460, pC59-153, and pYDC637. In contrast, just 1% of the reads showed similarity to *L. lactis* chromosomal sequences. Similarly, 95% of the sequences from the PCMA-60D library proved to be identical to sequences in mobile genetic elements from *E. faecium* and *E. faecalis*, such as pJH1, pRE25, pF856, plasmid 1, pVF18, and pVF4 (**Figure 2B**). In addition, 63% of the end-read sequences of clones from the MRS-3D library showed strong similarity to chromosomal sequences of *L. lactis*, while 37% showed similarity to plasmids from *L. lactis* or other LAB species, such as pEps352 (16%) and pIBB-JZK (11%) from *L. lactis*, p5753cB (5%) from *E. faecium*, and pKLC4 (5%) from *Leuconostoc carnosum* (**Figure 2C**). Surprisingly, all end-read sequences of the MRS-60D library were identical to the sequence of plasmid pF856 from *E. faecium* (**Figure 2D**).

Clones from each library were clustered into groups according to their sequence: PCMA-3D (25 groups), PCMA-60D (13 groups), MRS-3D (5 groups), and MRS-60D (4 groups). DNA from one clone from each of the groups was screened for the presence of tetracycline resistance genes via specific PCR amplification assays.

### Screening of Clones for Tetracycline Resistance Genes

Positive amplification for tetracycline resistance genes was obtained for all of the above 47 clones. A single tetracycline resistance gene was detected in each of the clones analyzed. In total, four different genes, [*tet*(A), *tet*(L), *tet*(M), and *tet*(S)], were identified in the four fosmid libraries. The *tet*(A) gene was the only gene detected in the 25 clones derived from the PCMA-3D library (**Figure 2**). In contrast, *tet*(S) and *tet*(M) were identified among the five clones from the MRS-3D library (three and two times, respectively). Finally, *tet*(L) was the only gene detected among the 17 clones from the two libraries at day 60 of manufacture (PCMA-60D and MRS-60D).

### Whole Sequence Analysis of Six Tetracycline-Resistant Fosmid Clones

**FIGURE 3:**
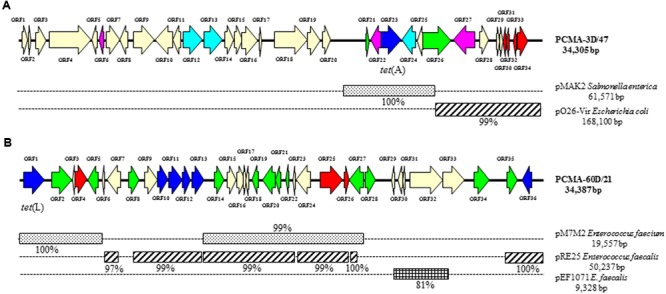
**Diagram of the genetic organization of two sequenced fosmid clones involved in tetracycline resistance obtained from PCMA plates.** Clones PCMA-3D/47 **(A)** and PCMA-60/21 **(B)**. Nucleotide sequence homology with known plasmids (on the right) and its percentage of identity is depicted below as black boxes. Color code of the different open reading frames (ORFs): Dark blue, antibiotic resistance genes; light blue, ABC and multidrug transporters; red, genes involved in plasmid replication and control; green, transposase-, integrase-, mobilization, and conjugation-associated genes; purple, genes encoding transcription regulators; pale yellow, genes involved in other processes.

**FIGURE 4:**
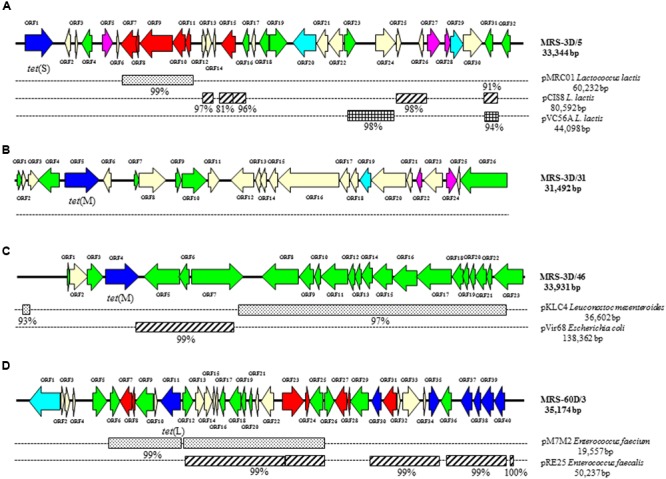
**Diagram of the genetic organization of four sequenced fosmid clones involved in tetracycline resistance obtained from MRS agar plates.** Clones MRS-3D/5 **(A)**, MRS-3D/31 **(B)**, MRS-3D/46 **(C)**, and MRS-60D/3 **(D)**. Nucleotide sequence homology with known plasmids (on the right) and its percentage of identity is depicted below as black boxes. Color code of the different ORFs: Dark blue, antibiotic resistance genes; light blue, ABC and multidrug transporters; red, genes involved in plasmid replication and control; green, transposase-, integrase-, mobilization, and conjugation associated genes; purple, genes encoding transcription regulators; pale yellow, genes involved in other processes.

Six representative tetracycline-resistant clones were selected for complete DNA sequencing. Two of these clones came from the PCMA-derived libraries (one each from the day 3 and day 60 libraries), and four from the MRS-derived libraries (three from the day 3 and one from the day 60 libraries). **Figures 3, 4** show the general features of the sequenced clones. In agreement with the sequencing results of the extremes, long segments of the sequenced clones showed extensive homology to plasmids from Gram-positive or Gram-negative bacteria. The exception was clone MRS-3D/31 (**Figure 4B**), the DNA and deduced protein sequences of which shared homology exclusively with chromosomal sequences from LAB species.

Clones PCMA-3D/47 (**Figure 3A**) and MRS-3D/46 (**Figure 4C**) carried sequences showing strong similarity to others in both Gram-positive and Gram-negative bacteria. Though DNA segments of plasmids from enterobacteria can be integrated into the genome of LAB species, the fosmid structures showed greater resemblance to chimera fragments of Gram-positive and Gram-negative origin formed by the ligation reaction.

#### Clone PCMA-3D/47

Thirty-four ORFs were identified in the 34,305 kbp sequence of clone PCMA-3D/47 (**Figure 3A** and Supplementary Table 1). A *tet*(A) gene was identified corresponding to ORF23, located in a 6 kbp DNA segment identical to plasmid pMAK2 from *Salmonella enterica*. The tetracycline resistance gene was flanked by insertion sequences related sequences coding for a relaxase (ORF21) and a transposase (ORF26). The right part (as shown in the **Figure 3A**) of the 5 kbp sequence of this clone showed 99% similarity to a segment of plasmid pO26-Vir from *E. coli*, while the sequence of more than 20 kbp to the left was identical to a chromosomal segment of *L. lactis*, in which the genes encoding aldose 1-epimerase, glucose-6-phosphate isomerase, valyl-tRNA synthetase, phosphopantetheine adenylyltransferase, and others, are encoded (Supplementary Table 1).

#### Clone PCMA-60D/21

A *tet*(L) gene coding for an efflux protein involved in tetracycline resistance was identified in ORF1 (**Figure 3B**). More than 90% of the sequence of this clone was identical or very similar to fragments from *Enterococcus* spp. plasmids. In agreement with a plasmid location, three ORFs (ORF4, ORF25, and ORF26) coding for proteins involved in plasmid replication homologous to those in enterococcal plasmids were identified. Further, 12 ORFs putatively coding for transposases, integrases and mobilization, plasmid-related proteins were identified (in green in **Figure 3B**). The whole clone may in fact correspond to a DNA segment from an enterococcal plasmid. In addition to *tet*(L), four other ORFs related to antibiotic resistance (ORFs 10, 11, 12, and 36) were identified (Supplementary Table 2). These might code for resistance to erythromycin, aminoglycosides and chloramphenicol.

#### Clone MRS-3D/5

A *tet*(S) gene encoding a ribosomal protection protein involved in tetracycline resistance was identified in ORF1 (**Figure 4A**). Segments of this clone showed strong similarity to others from several *L. lactis* plasmids, and the deduced proteins of most ORFs shared homology to plasmid-related proteins. Once again, many ORFs encoding transposases were identified over the sequence of this clone, along with others involved in plasmid replication and control (ORFs 7, 9, 10, 11, 12, and 15) (Supplementary Table 3).

#### Clone MRS-3D/31

A *tet*(M) gene was identified in ORF5 (**Figure 4B**); its nucleotide and deduced amino acid sequences were identical to those of the *tet*(M) gene in plasmid pSWS47 of *Staphylococcus epidermidis*. Generally speaking, the clone showed two halves of similar size (about 15 kbp each). The ORFs on the left half shared homology to *Enterococcus* and *Lactobacillus* sequences, while those on the right half were identical or very similar to others from *L. lactis*. Unlike all other clones, sequences in MRS-3D/31 showed no homology to plasmid-derived sequences. In spite of this, a large number of ORFs (in green in the figure) still encoded insertion sequence-related or mobilization-like proteins (Supplementary Table 4).

#### Clone MRS-3D/46

In this clone, tetracycline resistance is thought to have been provided by a *tet*(M) gene (ORF3) (**Figure 4C**). As shown in the latter figure, the gene is flanked by several transposase-related sequences. However, the origin of this resistance gene might be different to that in MRS-3D/3 since it proved to be identical to a resistance gene identified in a different species (*Staphylococcus aureus*) (Supplementary Table 5). MRS-3D/46 seems to be composed of several pieces. The right half of the clone, which encompasses more than 16 kbp, shows extensive homology to a segment of plasmid pKLC4 from *L. mesenteroides*, and encodes elements of a conjugative module (ORF8 through ORF23) (depicted in green in **Figure 4C**).

#### Clone MRS-60D/3

A *tet*(L) gene was identified in clone MRS-60D/3 (ORF11) (**Figure 4D**). In addition, some segments of this clone showed a structure resembling fragments of clone PCMA-60D/21 (**Figure 3B**). Both clones showed homology to the same enterococcal plasmids (pM7M2 and pER25) and shared genes and operons (Supplementary Tables 2, 6). Among the shared genes, those involved in erythromycin, and aminoglycoside resistance (ORFs 37, 38, 39, and 40 in this clone) were found.

### Tetracycline and Antibiotic Resistance of Fosmid Clones

The MICs of 16 antibiotics for *E. coli* EPI300-T1^R^ with and without the pCC1FOS vector, and harboring (separately) the six sequenced clones, were determined to assess the resistance conferred by the tetracycline resistance genes and other antibiotic resistance determinants. **Table 1** shows the values obtained. *E. coli* EPI300-T1^R^ harboring the vector and any of the clones was able to grow at the maximum analyzed concentrations of ampicillin, chloramphenicol, clindamycin, erythromycin, linezolid, penicillin G, quinupristin–dalfopristin, streptomycin, trimethoprim, and vancomycin. *E. coli* EPI300-T1R carries a mutation in the *rpsL* gene that confers streptomycin resistance, and chloramphenicol is the antibiotic resistance marker in pCC1FOS. All other resistances, must, therefore, be considered *E. coli* intrinsic resistances. As expected, all clones conferred resistance to tetracycline, although not to the same degree. The MICs of this antibiotic ranged from 16 to 64 μg mL^-1^, with the *tet*(L) gene, providing the greatest resistance. In the two clones for which aminoglycoside resistance determinants were detected (PCMA-60D/21 and MRS-60D/3), strong resistance to kanamycin and neomycin was also observed. Moderate resistance to gentamicin and rifampicin, and to rifampicin alone, was further provided by fosmids MRS-3D/5 and MRS-3D/46, respectively. However, the genes responsible for the latter resistances were not identified. Due to the vector carrying a chloramphenicol resistance gene, the involvement in resistance of a putative chloramphenicol acetyltransferase in clones PCMA-60D/21 (ORF36) and MRS-60D/3 (ORF30) could not be evaluated.

**Table 1:** Minimum Inhibitory Concentrations (MICs) of 16 antibiotics to the *Escherichia coli* host and selected Tc^r^ fosmid-clones as determined by microdilution in LB medium.

Antibiotic	MIC in μg mL^-1^					
	*E. coli* EPI300-T1^R^ pCC1FOS	PCMA-3D/47	PCMA-60D/21	MRS-3D/5	MRS-3D/31	MRS-3D/46	MRS-60D/3
Ampicillin^a^	>16	>6	>16	>16	>16	>16	>16
Penicillin G^a^	>16	>16	>16	>16	>16	>16	>16
Gentamicin	4	2	4	16	4	4	4
Kanamycin	8	8	**512**	32	8	8	**>1024**
Streptomycin^a,b^	>256	>256	>265	>256	>256	>256	>256
Neomycin	8	16	**256**	8	8	8	**>256**
Erythromycin^a^	>8	>8	>8	>8	>8	>8	>8
Clindamycin^a^	>16	>16	>16	>16	>16	>16	>16
Chloramphenicol^c^	>64	>64	>64	>64	>64	>64	>64
Tetracycline	0.5	**64**	**16**	**16**	**16**	**16**	**16**
Ciprofloxacin	<0.25	<0.25	<0.25	<0.25	<0.25	<0.25	<0.25
Linezolid^a^	>16	>16	>16	>16	>16	>16	>16
Quinupristin-dalfopristin^a^	>8	>8	>8	>8	>8	>8	>8
Trimethopim^a^	>64	>64	>64	>64	>64	>64	>64
Vancomycin^a^	>128	>128	>128	>128	>128	>128	>128
Rifampicin	2	2	4	8	2	8	4

*^*a*^*E. coli* EPI300-T1^*R*^ was able to grow at the maximum analyzed concentration of ampicillin, clindamycin, erythromycin, linezolid, penicillin G, quinupristin–dalfopristin, streptomycin, trimethoprim and vancomycin. ^*b*^*E. coli* EPI300-T1^*R*^ carries a mutation in the *rpsL* gene conferring streptomycin resistance. ^c^Chloramphenicol is the antibiotic resistance marker in pCC1FOS. Antibiotic resistances provided by (genes in) the clones are in bold.*

## Discussion

This study reports a functional metagenomic assessment of the diversity and evolution of tetracycline-resistant microbial populations and tetracycline resistance genes in an artisanal, blue-veined, cheese made of raw milk (with no starter culture added) at days 3 and 60 of manufacture. Tetracycline resistance was investigated since this antibiotic remains among the most used in livestock production worldwide (Pagel and Gautier, 2012). Further, a high prevalence of tetracycline-resistant bacteria has been reported for fermented foods, including dairy products (Gevers et al., 2003a; Flórez et al., 2005; Ammor et al., 2008; Li et al., 2011). Not surprisingly, many tetracycline resistance genes have been reported in LAB strains isolated from dairy settings (Devirgiliis et al., 2008), as well as directly in microbial DNA from artisanal and industrial cheeses (Devirgiliis et al., 2014; Flórez et al., 2014).

In the present work, a culturing step in the presence of tetracycline was introduced before microbial DNA extraction. Culturing in the presence of antibiotics is a common enrichment practice (Seville et al., 2009; Schmieder and Edwards, 2012), and it allowed large quantities of high molecular weight DNA to be extracted from tetracycline-resistant bacteria -DNA of the quality required for fosmid library construction. It also helped avoid the presence of contaminating DNA from eukaryotic cells from the food matrix (Devirgiliis et al., 2014), which is critical for a blue-cheese with large populations of yeast and molds, particularly at day 60 of ripening (Flórez et al., 2006). Tetracycline resistant microbial loads of around 10^6^ cfu g^-1^ cheese agrees well with previous results for artisanal and industrial cheeses (Devirgiliis et al., 2008; Li et al., 2011; Flórez and Mayo, 2015). The comparison of microbial counts at days 3 and 60 on media with and without tetracycline revealed a reduction in total mesophilic tetracycline-resistant populations over ripening, while the number of tetracycline-resistant LAB remained constant. This suggests that tetracycline-resistant populations other than LAB died during ripening, and that ripening does not promote growth of resistant LAB species.

Analysis of the tetracycline-resistant clones by restriction enzyme digestion and sequencing indicated a high diversity of large DNA inserts within each library, and large numbers of bacterial genomes. Compared to other metagenomic approaches based on high throughput sequencing (Mullany, 2014), functional metagenomics suffers the technical constraint of requiring expression of the genes of interest in *E. coli*. While most tetracycline-resistance determinants coding for ribosomal protection protein or efflux pumps have been shown to drive tetracycline resistance in this species (Thaker et al., 2010; van Hoek et al., 2011), that conferred by the present genes was significantly less strong than those reported elsewhere (Zycka-Krzesinska et al., 2015; Flórez et al., 2016). The expression of the tetracycline resistance genes in a single copy vector (pCC1FOS) must surely influence the resistance level.

A functional metagenomics investigation was selected given the possibility of finding genes coding for unknown (new) tetracycline resistance mechanisms that would otherwise be missed by PCR- and sequencing-based techniques (Diaz-Torres et al., 2003; Kazimierczak et al., 2009; Cheng et al., 2012; Mullany et al., 2012). However, despite the large genome coverage of the libraries, only four, well-known tetracycline resistance genes were encountered: *tet*(A), *tet*(L), *tet*(M), and *tet*(S). This might be due to a greater resistance pressure applied in this work during both the culturing and selection of clones as compared to that reported elsewhere (Kazimierczak et al., 2009; Cheng et al., 2012). The high resistance pressure aimed to avoid interference with the cloning of unrelated metabolic genes leading to antibiotic resistances (Thaker et al., 2010). The results suggest that the tetracycline resistome of Cabrales cheese is rather simple, although the presence of other tetracycline resistant determinants in minority populations, in populations that do not grow well under the culturing conditions used, or harbored in small-size plasmids that cannot be packaged into the head of lambda phages cannot be ruled out. A simple resistome is further supported by the absence in the cheese libraries of other tetracycline resistance genes frequently detected in dairy bacteria (and particularly so in LAB species) such as *tet*(W), *tet*(O), and *tet*(K) (Ammor et al., 2007; van Hoek et al., 2011).

To date, 46 tetracycline resistance genes classified into 11 classes have been characterized (Roberts and Schwarz, 2016). The majority of these classes (>60%) code for membrane-associated efflux proteins (Bryan et al., 2004), among which *tet*(A) has been recorded as the second most common tetracycline efflux pump in human and animal isolates, and the commonest in clinical and commensal isolates of *E. coli* (Sengeløv et al., 2003; Bryan et al., 2004). The presence of *tet*(A) of Gram-negative origin in all clones of the PCMA-3D library, and the homology of their end-read sequences to enterobacterial plasmids such as pO26-Vir, pC59-153, and pYDC637, suggests that the family Enterobacteriaceae (and *E. coli* in particular) are the majority tetracycline-resistant population at day 3. Further, *tet*(A) has been described in multi-resistant *E. coli* isolates from human and food animals (Fratamico et al., 2011; Wang et al., 2014; Lee et al., 2015). At day 3, *tet*(M) and *tet*(S) of Gram-positive origin were the only tetracycline resistance genes found among the clones from LAB (on MRS). All three genes were replaced by *tet*(L) also of Gram-positive origin in both libraries at day 60. Indeed, *tet*(L) was harbored by a single clone in the MRS-60D library. Sequence comparisons of the end-reads of clones carrying *tet*(M), *tet*(S), or *tet*(L) showed extensive homology to plasmids from *L. lactis* (pEps352 or pIBB-JZK) and *E. faecalis*/*E. faecium* (pRE25, pJH1, or pF856). In fact, all the end-read sequences of the clones from the MRS-60D library were identical to sequences of plasmid pF856 from *E. faecium* (Szakacs et al., 2014) -one of the most prevalent tetracycline resistance-carrying plasmids in this species. Altogether, these results indicate that *L. lactis* and *E. faecalis* and/or *E. faecium* account for the majority of the tetracycline-resistant LAB species, which agrees well with the culturing data of this and others studies on Cabrales cheese reporting large populations of Enterobacteriaceae to be present up to day 7 of manufacture, but which then decline thereafter (Flórez et al., 2006). In contrast, LAB species become the majority populations during ripening (Flórez et al., 2006). Since *Cabrales* is a raw-milk made cheese, in which large numbers of opportunistic and pathogenic species develop, it is not surprising that LAB species might acquire tetracycline resistance genes from them through horizontal transfer events.

In agreement with previous reports examining other environments (Kazimierczak et al., 2009; Mullany et al., 2012), the end-terminal sequences of the fosmid inserts showed more than 80% of them to carry nucleotide sequences with extensive homology to sequences of plasmid origin. This was further confirmed by analysis of the complete sequence of six clones, all but one of which (MRS-3D/31) harbored plasmid-derived sequences. It is well recognized that plasmids play a central part in the dissemination of antibiotic resistance genes between pathogens, and that they have contributed to the rapid development of multi-antibiotic resistance (Thomas, 2000; Bennett, 2008). Given the higher copy number of plasmid-borne genes (compared to those on the chromosome), and the mobile nature of plasmids, the antibiotic resistance genes harbored on the latter are thought to have the greatest chance of being horizontally transferred (Rossi et al., 2014). Indeed, most of the tetracycline resistance genes characterized to date in strains from dairy environments have been found in plasmids (Flórez et al., 2008, 2016; Devirgiliis et al., 2010), and plasmid-mediated transfer of antibiotic resistance between bacteria is well established (Flórez et al., 2008, 2016; Thomas, 2000; Rossi et al., 2014). Transfer is particularly likely when these genes are harbored on conjugative plasmids, as seems to be the case for *tet*(M) in the MRS-3D/46 clone. Though sophisticated molecular integron-like platforms for the capture of antibiotic resistance genes have not been reported in Gram-positive bacteria (Ravi et al., 2014), plasmid-derived sequences containing more than one antibiotic resistance gene were identified in two clones (PCMA-60D/21 and MRS-60D/3). It remains to be seen whether tetracycline resistance-carrying plasmids are maintained by the presence on the same molecules of niche-specific genes (such as those involved in lactose utilization, casein breakdown, etc.). Alternatively, co-selection could be driven by other antibiotic resistances, such as those encoded by aminoglycoside and putative chloramphenicol resistance genes in clones PCMA-60D/21 and MRS-60D/3.

Most tetracycline resistance genes on the sequenced clones were surrounded or flanked by ORFs encoding transposases, invertases, or mobilization proteins. The presence of these proteins around the tetracycline resistance regions is of special importance (Ammor et al., 2008; Mullany et al., 2012; Rossi et al., 2014) given their putative involvement in transference. Insertion sequences flanking antimicrobial resistance genes have been identified in the genomes of many bacteria. These elements can be organized as transposons carrying antibiotic resistances, as is commonly seen in clinical Enterobacteriaceae isolates (Nordmann et al., 2012; Johnson and Woodford, 2013). However, conjugative transposons such as Tn*916* and Tn*1549*, which are common in LAB, and which confer resistance upon these bacteria to antibiotics such as tetracycline, chloramphenicol, kanamycin, and erythromycin (Rossi et al., 2014), were never identified among the clones. Similarly, bacteriophage-related sequences, which are thought important in the transfer of antibiotic resistance in other ecosystems (Brown-Jaque et al., 2015; Calero-Cáceres and Muniesa, 2016), were also not observed.

## Conclusion

In conclusion, this functional metagenomic analysis provides a better understanding of the origin and evolution of tetracycline resistance genes in an artisanal dairy product. Further analysis of the resistome to the commonest antibiotics in clinical use, such as aminoglycosides, macrolides, quinolones, etc., could be of higher applied interest. This would provide an inventory of the resistance genes present while unearthing clues about their potential transfer mechanisms. Such information might be of use when estimating transfer risks. The same approach might be used to examine the resistome of other cheeses and indeed other fermented dairy products. All the tetracycline resistance genes characterized in this study resided on plasmids and/or were flanked by DNA sequences encoding putative insertion sequences, as well as mobilization- and conjugation-associated proteins. These mobile elements may contribute to the spread of tetracycline resistance genes among susceptible bacteria. Finally, raw-milk cheeses should be considered reservoirs of tetracycline resistance genes that might be horizontally transferred.

## Author Contributions

BM and AF conceived the study. AF and LV were involved in the experimental determinations. BM provided materials and resources. AF drafted the manuscript. BM made a critical revision of the manuscript. All authors reviewed and approved the final version.

## Conflict of Interest Statement

The authors declare that the research was conducted in the absence of any commercial or financial relationships that could be construed as a potential conflict of interest.

## Footnotes

1 https://blast.ncbi.nlm.nih.gov/Blast.cgi

2 http://www.ebi.ac.uk/zerbino/velvet/
