# Supplementary material for: A Functional Metagenomic Analysis of Tetracycline Resistance in Cheese Bacteria
Source: Front Microbiol. 2017 May 24;8:907. doi: 10.3389/fmicb.2017.00907 (PMC5442184; doi:10.3389/fmicb.2017.00907)
Supplement: Supplementary file 1 [file Table_1.docx]

**Supplementary Table 1.-** Analysis of the open reading frames (ORFs) identified in the fosmid clone PCMA-3D/47.

| ORF | 5’-end position^a^ | 3’-end position^a^ | % GC content | No. of aa^b^ | Known protein with the highest homology (microorganism) | % aa identity (identity length/total length) | GenBank Accession no. |
| --- | --- | --- | --- | --- | --- | --- | --- |
|  |  |  |  |  |  |  |  |
| ORF1 | 280 | 624 | 38 | 114 | Hypothetical protein (*Lactococcus lactis*) | 100% (114/114) | WP_010906360.1 |
| ORF2 | 663 | 911 | 33 | 82 | Aldose 1-epimerase (*L. lactis*) | 100% (82/82) | WP_003132639.1 |
| ORF3 | 1188 | 1844 | 36 | 218 | Fibronectin-binding protein (*L. lactis*) | 100% (218/218) | WP_017865187.1 |
| ORF4 | 2070 | 4742 | 39 | 890 | Valyl-tRNA synthetase (*L. lactis*) | 99% (882/890) | WP_015427197.1 |
| ORF5 | 5118 | 4786 | 31 | 110 | Hypothetical protein (*L. lactis*) | 100% (110/110) | WP_010906357.1 |
| ORF6 | 5563 | 5231 | 43 | 110 | Transcriptional regulator (*L. lactis*) | 100% (110/110) | WP_023164672.1 |
| ORF7 | 5736 | 6581 | 39 | 281 | NmrA family protein/Isoflavone reductase (*L. lactis*) | 100% (263/281) | WP_010906355.1 |
| ORF8 | 7121 | 6615 | 36 | 168 | Fe-S-oxidoreductase (*L. lactis*) | 100% (168/168) | WP_021214982.1 |
| ORF9 | 7465 | 8811 | 40 | 448 | Glucose-6-phosphate isomerase (*L. lactis*) | 100% (448/448) | WP_010906353.1 |
| ORF10 | 10013 | 8907 | 33 | 368 | ECF-type sigma factor negative effector (*L. lactis*) | 100% (368/368) | WP_023164670.1 |
| ORF11 | 10546 | 10037 | 32 | 169 | RNA polymerase sigma70 factor (*L. lactis*) | 100% (169/169) | WP_032947832.1 |
| ORF12 | 10719 | 11945 | 35 | 408 | lmrP MFS multidrug transporter (*L. lactis*) | 100% (408/408) | WP_023164669.1 |
| ORF13 | 12028 | 13248 | 35 | 406 | YwdA MFS transporter, multidrug resistance protein (*L. lactis*) | 100% (406/406) | WP_003132654.1 |
| ORF14 | 13419 | 13958 | 36 | 179 | rRNA methyltransferase (YwdG) (*L. lactis*) | 100% (179/179) | WP_015427191.1 |
| ORF15 | 14006 | 14503 | 34 | 165 | Phosphopantetheine adenylyltransferase (*L. lactis*) | 100% (165/165) | WP_010906348.1 |
| ORF16 | 14490 | 15518 | 39 | 342 | Peptidase S16 (*L. lactis*) | 99% (329/342) | WP_003132657.1 |
| ORF17 | 15639 | 15869 | 30 | 76 | Hypothetical protein (*L. lactis*) | 100% (76/76) | WP_023164667.1 |
| ORF18 | 16599 | 18722 | 32 | 707 | Radical SAM protein (*L. lactis*) | 100% (707/707) | WP_023164665.1 |
| ORF19 | 18715 | 19542 | 30 | 275 | Radical SAM protein (*L. lactis*) | 100% (275/275) | WP_010906344.1 |
| ORF20 | 19735 | 20232 | 28 | 165 | Hypothetical protein (*L. lactis*) | 99% (164/165) | WP_021214977.1 |
| ORF21 | 23313 | 23555 | 72 | 80 | Relaxase (Proteobacteria) | 100% (79/80) | WP_000844627.1 |
| ORF22 | 24264 | 23587 | 63 | 225 | Tet^r^ family transcriptional regulator (Proteobacteria) | 100% (225/225) | WP_000106218.1 |
| ORF23 | 24268 | 25542 | 63 | 424 | Tetracycline resistance protein (Proteobacteria) | 100% (424/424) | WP_000106218.1 |
| ORF24 | 26458 | 25574 | 59 | 294 | Multidrug transporter PecM (Proteobacteria) | 100% (294/294) | WP_000058717.1 |
| ORF25 | 26988 | 26596 | 61 | 130 | Isochorismatase (*Salmonella enterica*) | 100% (130/135) | WP_001214977.1 |
| ORF26 | 26947 | 28743 | 65 | 598 | Transposase (Gammaproteobacteria) | 100% (598/598) | WP_000470240.1 |
| ORF27 | 30307 | 28904 | 47 | 467 | Hypothetical YagA (*Escherichia coli*) | 99% (467/467) | WP_001396194.1 |
| ORF28 | 30604 | 31206 | 50 | 200 | Hypothetical protein (Enterobacteriaceae) | 100% (200/200) | WP_000517694.1 |
| ORF29 | 31441 | 31710 | 38 | 89 | Hypothetical protein (Enterobacteriaceae) | 100% (89/89) | WP_000973555.1 |
| ORF30 | 32182 | 31902 | 39 | 93 | Hypothetical protein (Enterobacteriaceae) | 100% (93/93) | WP_001105066.1 |
| ORF31 | 32563 | 32288 | 50 | 91 | Plasmid stabilization protein (Enterobacteriaceae) | 100% (91/91) | WP_000421257.1 |
| ORF32 | 32847 | 32563 | 56 | 94 | CopG family transcripitonal regulator (Enterobacteriaceae) | 100% (94/94) | WP_001178089.1 |
| ORF33 | 33057 | 32890 | 53 | 55 | Hypothetical protein (*Escherichia coli*) | 100% (55/55) | WP_001335629.1 |
|  |  |  |  |  |  |  |  |

^a^Including start and stop codons.

^b^aa, amino acids.
