# Supplementary material for: A Functional Metagenomic Analysis of Tetracycline Resistance in Cheese Bacteria
Source: Front Microbiol. 2017 May 24;8:907. doi: 10.3389/fmicb.2017.00907 (PMC5442184; doi:10.3389/fmicb.2017.00907)
Supplement: Supplementary file 2 [file Table_2.docx]

**Supplementary Table 2.-** Analysis of the open reading frames (ORFs) identified in the fosmid clone PCMA-60D/21.

| ORF | 5’-end position^a^ | 3’-end position | % GC content | No. of aa^b^ | Known protein with the highest homology (microorganism) | % aa identity (identity length/total length) | GenBank Accession no. |
| --- | --- | --- | --- | --- | --- | --- | --- |
|  |  |  |  |  |  |  |  |
| ORF1 | 19 | 1395 | 36 | 458 | Tetracycline resistance protein, TetL (Bacteria) | 99% (457/458) | WP_001574277.1 |
| ORF2 | 1896 | 3221 | 34 | 441 | Mob, recombinase (Bacillales) | 99% (440/441) | WP_002360708.1 |
| ORF3 | 3244 | 3420 | 30 | 58 | Hypothetical protein (Bacilli) | 98% (57/58) | WP_001795120.1 |
| ORF4 | 3445 | 4257 | 33 | 270 | Replication protein (*Enterococcus faecalis*) | 100% (270/270) | EFM83913.1 |
| ORF5 | 4977 | 4291 | 37 | 228 | Transposase (Bacilli) | 100% (228/228) | WP_002354485.1 |
| ORF6 | 5464 | 5255 | 38 | 69 | Omega protein (*Streptococcus* spp.) | 100% (69/69(71)) | WP_000260238.1 |
| ORF7 | 6459 | 5563 | 32 | 298 | Chromosome partitioning protein ParA (Firmicutes) | 100% (298/298) | WP_002334978.1 |
| ORF8 | 6996 | 7682 | 37 | 228 | Transposase (*Enterococcus faecium*) | 99% (227/228) | WP_013558432.1 |
| ORF9 | 8053 | 8922 | 34 | 289 | DNA polymerase (Firmicutes) | 100% (289/289) | WP_000228166.1 |
| ORF10 | 8903 | 9637 | 39 | 244 | SAM-dependent methyltransferase (Firmicutes) | 100% (244/244) | WP_000662263.1 |
| ORF11 | 9670 | 10578 | 36 | 302 | Aminoglycoside 6-adenylyltransferase (Firmicutes) | 100% (302/302) | WP_001255866.1 |
| ORF12 | 10575 | 11117 | 38 | 180 | Streptothricin acetyltransferase (*Staphylococcus epidermidis*) | 100% (179/180) | WP_000627290.1 |
| ORF13 | 11210 | 12004 | 45 | 264 | Aminoglycoside 3'-phosphotransferase (Bacteria) | 100% (264/264) | WP_001096887.1 |
| ORF14 | 12693 | 13379 | 37 | 228 | Transposase (*E. faecium*) | 99% (227/228) | WP_013558432.1 |
| ORF15 | 13577 | 14182 | 33 | 201 | Cell division protein Fic (Bacilli) | 100% (201/201) | WP_000599739.1 |
| ORF16 | 14189 | 14770 | 40 | 193 | DNA invertase Pin (*E. faecium*) | 99% (192/193) | WP_002321591.1 |
| ORF17 | 14884 | 15042 | 35 | 52 | Hypothetical protein (*E. faecium*) | 100% (52/52) | WP_002317484.1 |
| ORF18 | 15183 | 15023 | 30 | 53 | Hypothetical protein (*E. faecium*) | 100% (53/53) | WP_002317483.1 |
| ORF19 | 15665 | 15243 | 35 | 140 | Integrase (Bacilli) | 100% (140/140) | WP_000363099.1 |
| ORF20 | 16752 | 15916 | 38 | 278 | Transposase (*E. faecium*) | 99% (277/248) | WP_010730633.1 |
| ORF21 | 17117 | 16788 | 35 | 109 | Transposase (*E. faecium*) | 99% (108/109) | WP_002322074.1 |
| ORF22 | 17738 | 17484 | 36 | 84 | Transposase (Bacilli) | 100% (84/84) | WP_000199136.1 |
| ORF23 | 18206 | 17931 | 34 | 91 | Putative PrgO protein (*E. faecalis*) | 100% (91/91) | WP_001196543.1 |
| ORF24 | 19131 | 18178 | 35 | 317 | Chromosome partitioning ATPase (Bacilli) | 100% (317/317) | WP_000429439.1 |
| ORF25 | 19743 | 21236 | 36 | 497 | Plasmid replication protein RepR (Bacilli) | 100% (497/497) | WP_000947691.1 |
| ORF26 | 21350 | 21667 | 37 | 105 | Replication control protein PrgN (*Enterococcus* spp.) | 100% (105/105) | WP_002287239.1 |
| ORF27 | 22650 | 21691 | 36 | 319 | Integrase (*E. faecalis*) | 100% (319/319) | EPR45135.1 |
| ORF28 | 22732 | 23418 | 37 | 228 | Transposase (*E. faecium*) | 99% (227/228) | WP_013558432.1 |
| ORF29 | 24717 | 24550 | 28 | 55 | Hypothetical protein (*E. faecalis*) | 100% (55/55) | EFM82099.1 |
| ORF30 | 25238 | 24915 | 30 | 107 | Hypothetical protein (*E. faecalis*) | 100% (107/107) | WP_002402450.1 |
| ORF31 | 25460 | 25260 | 40 | 66 | Bacteriocin-type signal peptide protein (*E. faecalis*) | 100% (66/66) | WP_002402451.1 |
| ORF32 | 25631 | 27781 | 33 | 716 | Bacteriocin ABC transporter-ATP-binding protein (*E. faecalis*) | 100% (716/716) | WP_002402452.1 |
| ORF33 | 27769 | 29166 | 32 | 465 | Bacteriocin secretion accessory protein (*E. faecalis*) | 100% (465/465) | EFM82103.1 |
| ORF34 | 29845 | 30855 | 37 | 336 | Plasmid recombination enzyme (*E. faecalis*) | 100% (336/336) | WP_002402455.1 |
| ORF35 | 31996 | 32682 | 37 | 228 | Transposase (*E. faecium*) | 99% (227/228) | WP_013558432.1 |
| ORF36 | 33675 | 33028 | 24 | 215 | Chloramphenicol acetyltransferase (Bacilli) | 100% (215/215) | WP_002331392.1 |
|  |  |  |  |  |  |  |  |

^a^Including start and stop codons.

^b^aa, amino acids.
