# Supplementary material for: A Functional Metagenomic Analysis of Tetracycline Resistance in Cheese Bacteria
Source: Front Microbiol. 2017 May 24;8:907. doi: 10.3389/fmicb.2017.00907 (PMC5442184; doi:10.3389/fmicb.2017.00907)
Supplement: Supplementary file 3 [file Table_3.docx]

**Supplementary Table 3.-** Analysis of the open reading frames (ORFs) identified in the fosmid clone MRS-3D/5.

| ORF | 5’-end position^a^ | 3’-end position^a^ | % GC content | No. of aa^b^ | Known protein with the highest homology (microorganism) | % aa identity (identity length/total length) | GenBank Accession no. |
| --- | --- | --- | --- | --- | --- | --- | --- |
|  |  |  |  |  |  |  |  |
| ORF1 | 360 | 2301 | 33 | 646 | Tetracycline resistance protein (*Lactococcus garvieae*) | 100% (646/646) | YP_001798653.1 |
| ORF2 | 3428 | 3069 | 37 | 119 | Hypothetical protein pK214_p30 (*Lactococcus lactis* subsp. *lactis*) | 100% (119/119) | YP_001429540.1 |
| ORF3 | 3905 | 4135 | 37 | 76 | Hypothetical protein pK214_p29 (*L. lactis* subsp. *lactis*) | 100% (76/76) | YP_001429539.1 |
| ORF4 | 4908 | 4189 | 37 | 239 | IS1216 transposase (*Enterococcus faecalis*) | 99% (225/228) | NP_816937.1 |
| ORF5 | 5591 | 6310 | 29 | 239 | Hypothetical protein (transcriptional regulator, MarR family/Acetyltransferase | No homology in databases | - |
| ORF6 | 6661 | 6828 | 28 | 55 | Hypothetical protein | No homology in databases |  |
| ORF7 | 7906 | 6839 | 28 | 355 | LtrC (*L. lactis*) | 99% 355(355/355) | WP_010890638.1 |
| ORF8 | 8118 | 7909 | 30 | 69 | Hypothetical protein (*L. lactis*) | 100% (69/69) | WP_010890684.1 |
| ORF9 | 10298 | 8121 | 28 | 725 | Protein TrsI (*L. lactis*) | 99% (725/725) | WP_010890637.1 |
| ORF10 | 11150 | 10314 | 32 | 278 | Protein TrsL (*L. lactis*) | 99% (278/278) | WP_010890636.1 |
| ORF11 | 11560 | 11165 | 27 | 131 | Hypothetical protein pMRC01_018 (*L. lactis*) | 100% (131/110) | NP_047303.1 |
| ORF12 | 12284 | 12565 | 24 | 93 | Hypothetical protein (*L. lactis*) | 100% (79/93) | WP_031560353.1 |
| ORF13 | 12571 | 12948 | 25 | 125 | Hypothetical protein (*L. lactis* subsp. *cremoris*) | 99% (125/125) | EUN33410.1 |
| ORF14 | 13325 | 13083 | 25 | 80 | Hypothetical protein (*L. lactis*) | 100% (80/84) | WP_021214641.1 |
| ORF15 | 14576 | 13563 | 34 | 337 | RepB protein (*L. lactis*) | 99% (312/383) | WP_021214640.1 |
| ORF16 | 15465 | 15040 | 37 | 141 | Resolvase (*L. lactis* subsp. *lactis*) | 99% (141/178) | EQC89294.1 |
| ORF17 | 15870 | 15592 | 28 | 92 | Hypothetical protein (*L. lactis*) | 98% (92/92) | WP_021214854.1 |
| ORF18 | 16776 | 16156 | 39 | 206 | Transposase IS982 (*L. lactis*) | 97% (188/296) | WP_023349191.1 |
| ORF19 | 16828 | 18036 | 45 | 402 | Transposase (IS10, IS4, TN10) (Multispecies) | 100% (402/402) | WP_001339197.1 |
| ORF20 | 19977 | 18394 | 32 | 527 | ABC transporter permease protein (*L. lactis*) | 100% (526/526) | WP_021037031.1 |
| ORF21 | 20719 | 19955 | 37 | 254 | 3-Dehydroquinate dehydratase (*L. lactis*) | 100% (254/254) | WP_011835457.1 |
| ORF22 | 21741 | 20842 | 36 | 299 | Permease of the drug/metabolite superfamily (*L. lactis*) | 99% (299/299) | WP_021214755.1 |
| ORF23 | 21891 | 22637 | 40 | 248 | Transposase IS982 (*L. lactis*) | 98% (247/296) | WP_023349191.1 |
| ORF24 | 23973 | 25295 | 36 | 440 | ImpB/MucB/SamB polymerase family protein (*L. lactis*) | 100% (440/488) | WP_014570266.1 |
| ORF25 | 25358 | 25687 | 32 | 109 | Hypothetical protein (*L. lactis*) | 100% (109/131) | WP_014570265.1 |
| ORF26 | 27196 | 26861 | 34 | 111 | Peptide methionine sulfoxide reductase (*L. lactis* subsp. *lactis*) | 99% (111/111) | ESK78514.1 |
| ORF27 | 27475 | 28311 | 31 | 278 | AraC family transcriptional regulator (*L. lactis*) | 98% (222/278) | WP_032398644.1 |
| ORF28 | 28626 | 28991 | 33 | 121 | Transcriptional regulator, GntR family (*L. lactis*) | 100% (121/121) | WP_017864521.1 |
| ORF29 | 28988 | 29878 | 32 | 294 | ABC transporter (*L. lactis*) | 99% (294/294) | WP_032398668.1 |
| ORF30 | 29865 | 31151 | 30 | 428 | Hypothetical protein (*L. lactis*) | 99% (428/428) | WP_021214708.1 |
| ORF31 | 31857 | 31291 | 35 | 188 | Resolvase/integrase Bin (*L. lactis* subsp. *cremoris*) | 96% (188/189) | AFK83769.1 |
| ORF32 | <32596 | 32171 | 36 | 152 | IS6 family transposase (*Enterococcus faecium*) | 100% (152/152) | WP_010728590.1 |
|  |  |  |  |  |  |  |  |

^a^Including start and stop codons.

^b^aa, amino acids.

< 5' partial feature
