# Supplementary material for: A Functional Metagenomic Analysis of Tetracycline Resistance in Cheese Bacteria
Source: Front Microbiol. 2017 May 24;8:907. doi: 10.3389/fmicb.2017.00907 (PMC5442184; doi:10.3389/fmicb.2017.00907)
Supplement: Supplementary file 4 [file Table_4.docx]

**Supplementary Table 4.-** Analysis of the open reading frames (ORFs) identified in the fosmid clone MRS-3D/31.

| ORF | 5’-end position^a^ | 3’-end position^a^ | % GC content | No. of aa^b^ | Known protein with the highest homology (microorganism) | % aa identity (identity length/total length) | GenBank Accession no. |
| --- | --- | --- | --- | --- | --- | --- | --- |
|  |  |  |  |  |  |  |  |
| ORF1 | 807 | 1181 | 46 | 124 | Conjugative transposon protein (*Enterococcus faecium*) | 100% (116/223) | WP_002328815.1 |
| ΔORF2 | <1216 | 1539 | 49 | 107 | Hypothetical protein (*Enterococcus malodoratus*) | 79% (107/283) | WP_010742004.1 |
| ΔORF3 | <1699 | 2556 | 52 | 285 | Short chain dehydrogenase (*Paenibacillus alvei*) | 50% (283/299) | WP_021258232.1 |
| ORF4 | 4127 | 2919 | 54 | 402 | Transposase of Tn10 (*Shigella flexneri*) | 100% (402/402) | NP_052934 |
| ORF5 | 4572 | 6506 | 49 | 644 | Tetracycline resistance protein Tet(M) in plasmid pSWS47 (*Staphylococcus epidermidis*) | 100% (644/644) | YP_008719892.1 |
| ORF6 | 7262 | 6795 | 53 | 155 | Stress induced DNA binding protein (*Lactobacillus plantarum*) | 100% (155/155) | YP_003064085.1 |
| ΔORF7 | <7818 | >9525 | 45 | 94 | Integrase (*Lactobacillus pobuzihii*) | 82% (94/195) | WP­_017868399 |
| ORF8 | 9047 | 10546 | 44 | 499 | Hypothetical protein | No homology in databases | - |
| ORF9 | 11152 | 11475 | 41 | 107 | Plasmid mobilization protein MobC (*Lactobacillus paracasei*) | 45% (107/112) | EPC69208 |
| ORF10 | 11454 | 12842 | 39 | 462 | Plasmid mobilization protein MobA (*L. paracasei*) | 41% (348/445) | EPC69207.1 |
| ORF11 | 12839 | 13504 | 42 | 221 | Hypothetical protein | No homology in databases | - |
| ORF12 | 15669 | 14311 | 54 | 452 | Beta-lactamase-like protein (*Lactococcus lactis* subsp. *lactis*) | 100% (452) | WP_003132173.1 |
| ORF13 | 16121 | 15798 | 54 | 107 | Hypothetical protein (*L. lactis* subsp. *lactis*) | 100% (107) | NP_266173.1 |
| ORF14 | 16495 | 16121 | 56 | 124 | Septum formation initiator (*L. lactis* subsp. *lactis*) | 100% (124) | YP_005867419.1 |
| ORF15 | 17382 | 16912 | 47 | 156 | Hypothetical protein (*Enterococcus gallinarum*) | 99% (156) | ERE64735.1 |
| ORF16 | 21026 | 17541 | 49 | 1161 | Transcription-repair coupling factor (*L. lactis* subsp. *lactis*) | 99% (1161) | YP_005867416.1 |
| ORF17 | 21593 | 21027 | 49 | 188 | Peptidyl-tRNA hydrolase (*L. lactis* subsp. *cremoris*) | 100% (188) | YP_001031377.1 |
| ORF18 | 22081 | 21611 | 52 | 156 | Hypothetical protein (*L. lactis* subsp. *lactis*) | 100% (156) | YP_005867414.1 |
| ORF19 | 22911 | 22276 | 49 | 211 | ABC transporter ATP-binding protein (*L. lactis* subsp. *lactis*) | 100% (211) | YP_005867413.1 |
| ORF20 | 24904 | 22913 | 47 | 663 | Hypothetical protein (*L. lactis* subsp. *lactis*) | 99% (613/663) | WP_003132163.1 |
| ORF21 | 25261 | 24917 | 49 | 114 | Hypothetical protein (*L. lactis* subsp. *lactis*) | 100% (114) | YP_005867411.1 |
| ORF22 | 25839 | 25516 | 50 | 107 | Cro/CI family transcriptional regulator (*L. lactis* subsp. *lactis*) | 99% (107) | YP_005867410.1 |
| ORF23 | 27066 | 25951 | 49 | 371 | GTP-dependent nucleic acid-binding protein (*L. lactis* subsp. *lactis*) | 100% (371) | YP_005867409.1 |
| ORF24 | 27348 | 27905 | 54 | 185 | Transcriptional regulator (*L. lactis* subsp. *lactis*) | 99% (185) | NP_266161.1 |
| ORF25 | 28115 | 27915 | 37 | 66 | Hypothetical protein (*L. lactis* subsp. *lactis*) | 100% (66/66) | WP_003132155.1 |
| ΔORF26 | <31451 | 28152 | 32 | 988 | Helicase-exonuclease AddAB, subunit AddA (*L. lactis*) | 100% (988/1203) | WP_014570317.1 |
|  |  |  |  |  |  |  |  |

^a^Including start and stop codons.

^b^aa, amino acids.

< 5' partial feature

< 3' partial feature
