# Supplementary material for: A Functional Metagenomic Analysis of Tetracycline Resistance in Cheese Bacteria
Source: Front Microbiol. 2017 May 24;8:907. doi: 10.3389/fmicb.2017.00907 (PMC5442184; doi:10.3389/fmicb.2017.00907)
Supplement: Supplementary file 5 [file Table_5.docx]

**Supplementary Table 5.-** Analysis of the open reading frames (ORFs) identified in the fosmid clone MRS-3D/46.

| ORF | 5’-end position^a^ | 3’-end position^a^ | % GC content | No. of aa^b^ | Known protein with the highest homology (microorganism) | % aa identity (identity length/total length) | GenBank Accession no. |
| --- | --- | --- | --- | --- | --- | --- | --- |
|  |  |  |  |  |  |  |  |
| ORF1 | 6569 | 6108 | 57 | 153 | Resolvase family site-specific recombinase (*Pediococcus claussenii*) | 96% (97/186) | YP_005353081.1 |
| ORF2 | 6797 | 7846 | 50 | 349 | Hypothetical protein (*Lactobacillus plantarum*) | 99% (337/337) | WP_011117013.1 |
| ORF3 | 7797 | 8723 | 46 | 308 | Conjugal transfer protein (*Lactobacillus oryzae*) | 100% (308/309) | WP_034529644.1 |
| ORF4 | 9085 | 11019 | 50 | 644 | Tetracycline resistance protein TetM (*Staphylococcus aureus*) | 100% (644/644) | YP_005743777.1 |
| ORF5 | 13493 | 11397 | 52 | 698 | Transposase (*Escherichia coli*) | 100% (698/689) | WP_000422420.1 |
| ORF6 | 14141 | 13509 | 48 | 210 | Putative transposon resolvase (*Escherichia coli*) | 100% (210/210) | WP_012775882.1 |
| ORF7 | 14224 | 17232 | 51 | 1002 | Transposase (*Escherichia coli*) | 100% (1002/1002) | WP_001143750.1 |
| ORF8 | 20893 | 18725 | 57 | 722 | DNA topoisomerase (Lactobacillales) | 100% (722/722) | WP_015007093.1 |
| ORF9 | 21749 | 20913 | 47 | 278 | Conjugal transfer protein TrsL (Lactobacillales) | 99% (278/278) | WP_015007094.1 |
| ORF10 | 22161 | 21766 | 58 | 131 | Hypothetical protein (Lactobacillales) | 98% (131/131) | WP_015007095.1 |
| ORF11 | 23700 | 22174 | 53 | 508 | Conjugal transfer protein (Lactobacillales) | 100% (509/514) | WP_015007096.1 |
| ORF12 | 24155 | 23697 | 53 | 152 | Conjugal transfer protein (Lactobacillales) | 98% (152/152) | WP_015007097.1 |
| ORF13 | 24526 | 24161 | 52 | 121 | Thioredoxin (Lactobacillales) | 98% (121/121) | WP_015007098.1 |
| ORF14 | 25133 | 24516 | 57 | 205 | Hypothetical protein (Lactobacillales) | 98% (205/205) | WP_015007099.1 |
| ORF15 | 26309 | 25146 | 57 | 387 | Peptidoglycan hydrolase (Lactobacillales) | 96% (387/387) | WP_015007100.1 |
| ORF16 | 27720 | 26302 | 56 | 472 | Conjugal transfer protein (Lactobacillales) | 98% (472/472) | WP_015007064.1 |
| ORF17 | 29737 | 27713 | 54 | 674 | Conjugal transfer protein (Lactobacillales) | 98% (672/672) | WP_015007065.1 |
| ORF18 | 30412 | 29750 | 54 | 220 | Conjugal transfer protein (Lactobacillales) | 99% (217/217) | WP_015007066.1 |
| ORF19 | 30734 | 30384 | 50 | 116 | Conjugal transfer protein (Lactobacillales) | 97% (116/116) | WP_015007067.1 |
| ORF20 | 31111 | 30767 | 48 | 114 | Conjugal transfer protein (Lactobacillales) | 98% (114/114) | WP_015007068.1 |
| ORF21 | 31741 | 31118 | 57 | 207 | Hypothetical protein (Lactobacillales) | 97% (208/208) | WP_015007069.1 |
| ORF22 | 32094 | 31774 | 53 | 106 | Hypothetical protein (Lactobacillales) | 96% (106/106) | WP_015007070.1 |
| ORF23 | 33856 | 32177 | 55 | 559 | MobA/MobL family mobilization protein (Lactobacillales) | 97% (468/468) | WP_015007071.1 |
|  |  |  |  |  |  |  |  |

^a^Including start and stop codons.

^b^aa, amino acids.
