# Supplementary material for: A Functional Metagenomic Analysis of Tetracycline Resistance in Cheese Bacteria
Source: Front Microbiol. 2017 May 24;8:907. doi: 10.3389/fmicb.2017.00907 (PMC5442184; doi:10.3389/fmicb.2017.00907)
Supplement: Supplementary file 6 [file Table_6.docx]

**Supplementary Table 6.-** Analysis of the open reading frames (ORFs) identified in the fosmid clone MRS-60D/3.

| ORF | 5’-end position^a^ | 3’-end position^a^ | % GC content | No. of aa^b^ | Known protein with the highest homology (microorganism( | % aa identity (identity length/total length) | GenBank Accession no. |
| --- | --- | --- | --- | --- | --- | --- | --- |
|  |  |  |  |  |  |  |  |
| ORF1 | 3216 | 1066 | 33 | 716 | ABC transporter ATP-binding protein (*Enterococcus faecalis*) | 100% (719/719) | WP_002402452.1 |
| ORF2 | 3387 | 3587 | 40 | 66 | Signal peptide protein (*E. faecalis*) | 100% (66/66) | WP_002402451.1 |
| ORF3 | 3609 | 3932 | 30 | 107 | Hypothetical protein (*E. faecalis*) | 100% (107/107) | WP_002402450.1 |
| ORF4 | 4130 | 4297 | 28 | 55 | Hypothetical protein (*E. faecalis*) | 100% (55/55) | EFM82099.1 |
| ORF5 | 5553 | 6569 | 54 | 338 | IS5 transposase and transactivator (*Escherichia coli*) | 100% (338/338) | NP_414793.1 |
| ORF6 | 6767 | 7453 | 37 | 228 | Transposase (Bacilli) | 100% (228/228) | WP_002354485.1 |
| ORF7 | 8299 | 7487 | 33 | 270 | Replication protein (*E. faecalis*) | 100% (270/270) | EFM83913.1 |
| ORF8 | 8500 | 8324 | 30 | 58 | Hypothetical protein (*Staphylococcus aureus*) | 100% (58/58) | YP_039505.1 |
| ORF9 | 9848 | 8523 | 34 | 441 | Mobilization protein (*Bacillus cereus*) | 99% (441/441) | NP_043522.1 |
| ORF10 | 9892 | 10065 | 44 | 57 | Hypothetical protein (*Enterococcus faecium*) | 98% (57/57) | YP_004849395.1 |
| ORF11 | 11725 | 10349 | 36 | 458 | Tetracycline resistance protein TetL (*Streptococcus agalactiae*) | 99% (458/458) | NP_040422.1 |
| ORF12 | 11949 | 12635 | 37 | 228 | Transposase (Bacilli) | 100% (228/228) | WP_002354485.1 |
| ORF13 | 12833 | 13438 | 34 | 201 | Cell division protein Fic (Bacilli) | 100% (201/201) | WP_000599739.1 |
| ORF14 | 13445 | 14026 | 40 | 193 | DNA invertase Pin (*E. faecium*) | 99% (193/193) | WP_002321591.1 |
| ORF15 | 14140 | 14298 | 35 | 52 | Hypothetical protein (*E. faecium*) | 100% (52/52) | WP_002317484.1 |
| ORF16 | 14439 | 14278 | 30 | 53 | Hypothetical protein (*E. faecium*) | 100% (53/53) | WP_002317483.1 |
| ORF17 | 14921 | 14499 | 35 | 140 | Transposase (*E. faecium*) | 100% (140/140) | YP_004849412.1 |
| ORF18 | 16008 | 15172 | 38 | 278 | Transposase (*E. faecium*) | 99% (278/278) | WP_010730633.1 |
| ORF19 | 16373 | 16044 | 35 | 109 | Transposase (*E. faecium*) | 99% (109/109) | WP_002322074.1 |
| ORF20 | 16994 | 16740 | 36 | 84 | Transposase (Bacilli) | 100% (84/84) | WP_000199136.1 |
| ORF21 | 17462 | 17187 | 34 | 91 | Hypothetical protein (Bacilli) | 100% (91/91) | WP_001196543.1 |
| ORF22 | 18387 | 17434 | 35 | 317 | Chromosome partitioning ATPase (Bacilli) | 100% (317/317) | WP_000429439.1 |
| ORF23 | 18999 | 20491 | 36 | 497 | RepR protein (Bacilli) | 100% (497/497) | WP_000947691.1 |
| ORF24 | 20606 | 20923 | 37 | 105 | Hypothetical protein (*Enterococcus* spp.) | 100% (105/105) | WP_002287239.1 |
| ORF25 | 21906 | 20947 | 36 | 319 | Integrase (Bacilli) | 100% (319/319) | WP_000222573.1 |
| ORF26 | 21988 | 22674 | 37 | 228 | Transposase (Bacilli) | 100% (228/228) | WP_002354485.1 |
| ORF27 | 23520 | 22708 | 33 | 270 | Replication protein (*E. faecalis*) | 100% (270/270) | EFM83913.1 |
| ORF28 | 23721 | 23545 | 30 | 58 | Hypothetical protein (Bacilli) | 98% (58/58) | WP_001795120.1 |
| ORF29 | 25069 | 23744 | 34 | 441 | Recombinase (Bacillales) | 100% (441/441) | WP_002360708.1 |
| ORF30 | 25958 | 25311 | 24 | 215 | Chloramphenicol acetyltransferase (Bacilli) | 100% (215/215) | WP_002331392.1 |
| ORF31 | 27026 | 26088 | 32 | 312 | Replication initiation protein (Lactobacillales) | 100% (312/312) | WP_002331393.1 |
| ORF32 | 26987 | 27160 | 30 | 57 | Hypothetical protein (*E. faecium*) | 98% (57/57) | WP_002321504.1 |
| ORF33 | 27526 | 28734 | 34 | 402 | Hypothetical protein (*E. faecalis*) | 100% (402/402) | WP_002331394.1 |
| ORF34 | 28868 | 29137 | 33 | 89 | Omega transcriptional repressor (Bacilli) | 99% (89/89) | WP_002368882.1 |
| ORF35 | 29366 | 30103 | 33 | 245 | ErmB, rRNA adenine N-6-methyltransferase (Bacteria) | 100% (245/245) | WP_002292226.1 |
| ORF36 | 30916 | 30230 | 37 | 228 | Transposase (Bacilli) | 100% (228/228) | WP_002354485.1 |
| ORF37 | 32399 | 31605 | 45 | 264 | Neomycin-kanamycin phosphotransferase type III, APH(3'(III (Bacteria) | 100% (264/264) | WP_001096887.1 |
| ORF38 | 33034 | 32492 | 38 | 180 | Streptothricin acetyltransferase, Sat4 (*Staphylococcus epidermidis*) | 99%(180/180) | YP_187540.1 |
| ORF39 | 33939 | 33031 | 36 | 302 | Streptomycin aminoglycoside 6-adenyltransferase; AadE (Firmicutes) | 100% (302/302) | WP_001255866.1 |
| ORF40 | 34706 | 33972 | 39 | 244 | SAM-dependent methyltransferase of pRUM_p17 (*E. faecium*) | 100% (244/244) | NP_863160.1 |
|  |  |  |  |  |  |  |  |

^a^Including start and stop codons.

^b^aa, amino acids.
